# Changes Over Time in Post-traumatic Stress Disorder Among Children Who Survived the 2008 Wenchuan Earthquake and Predictive Variables

**DOI:** 10.3389/fpsyt.2021.691765

**Published:** 2021-09-22

**Authors:** Ying Chen, Chow Lam, Hong Deng, Kam Ying Ko

**Affiliations:** ^1^Mental Health Center, West China Hospital, Sichuan University, Chengdu, China; ^2^Department of Psychology, Illinois Institute of Technology, Chicago, IL, United States; ^3^Hong Kong Youth Foundation, Hong Kong, China

**Keywords:** post-traumatic stress disorder, children, earthquake, predictive variables, cognitive behavioral therapy

## Abstract

**Background:** This study examines changes over time in post-traumatic stress disorder (PTSD) among children who survived the 2008 Wenchuan earthquake and the relevant predictive variables.

**Methods:** A total of 203 children and adolescents were investigated 24 months after the earthquake, and 151 children and adolescents completed the 1-year follow-up study. Participants completed the Children's Revised Impact of Event Scale (CRIES-13), the Center for Epidemiologic Studies Depression Scale (CES-D) and the Connor-Davidson Resilience Scale (CD-RISC). Hierarchical linear regression analysis was used to evaluate the predictors of changes in PTSD severity.

**Results:** Eighty percent of the children still had some PTSD symptoms 2 years after the Wenchuan earthquake, and 66.25% of the children had symptoms that lasted 3 years. In the model predicting PTSD symptom severity, the loss of family members and child burial explained a significant 21.9% of the variance, and depression explained a significant 16.7% of the variance. In the model predicting changes in PTSD severity, the change scores for resilience and depression explained a significant 18.7% of the variance, and cognitive behavioral therapy (CBT) explained a significant 33.6% of the variance.

**Conclusions:** PTSD symptoms in children and adolescents can persist for many years after trauma. In addition to using psychological interventions to improve PTSD symptoms, improvements in depression and resilience should also be considered.

## Introduction

On May 12, 2008, a Richter Scale 8.0 earthquake turned entire mountain towns in Sichuan Province into rubble, claiming the lives of 69,227 people, injuring 394,643, and leaving 17,923 missing in the ruins. As the earthquake occurred in the afternoon, students were in school classes. Many adolescents were exposed to horrific sights and sounds. Many of their friends and classmates were injured or died.

It has been found that the mental health of adolescents is seriously impacted by earthquakes (1, 2); most notably, many adolescents develop post-traumatic stress disorder (PTSD) (3, 4). The findings of many studies have shown that the proportion of children who develop PTSD after a traumatic event ranges from 10 to 70% (5–8). Ying et al. found that the prevalence of PTSD among children 6 months and 1 year after the Wenchuan earthquake was 11.2 and 13.4%, respectively (6). Fu et al. also showed that 14.1% of college students had been diagnosed with PTSD within 1 year after the Wenchuan earthquake (7). For children and adolescents who survived the earthquake, symptoms of PTSD may persist for a long time, as found in Jia et al.'s study, which showed that the 10.7% to 12.4% prevalence of PTSD did not significantly change from 15 months to 36 months after the earthquake (8).

Recently, various studies have summarized several risk factors for PTSD among children and adolescents who were survivors of natural disasters. Many studies have examined the relationships between demographic characteristics and PTSD. Age and gender are thought to play an important role in the development of PTSD in children who develop after a disaster (6, 9, 10). Some studies have indicated that younger children have less capacity to deal with trauma, which may result in them having more PTSD symptoms than older children (11). However, other studies have shown that older children had more severe symptoms than younger children (12). A meta-analysis of risk factors for PTSD indicated that age may moderate the relationship between PTSD and gender and that females have a significantly increased risk of developing PTSD with increasing age (13). Carmass et al.'s study suggested inconsistent results that younger females had more PTSD symptoms than older females (14). Objective trauma characteristics, such as the degree of exposure (in terms of distance from the seismic epicenter), burial and injury, witnessing death, and the loss of family members, are also related to the severity of PTSD (13, 14). For example, individuals who were closer to the seismic epicenter at the time of the earthquake had higher PTSD symptom levels (14). Adolescents who had lost family members had more PTSD symptoms than those who did not. Previous studies also indicated a close association between PTSD and depression (15). Major depression may facilitate the development of PTSD after a disaster; the presence of PTSD may also increase the risk of depression. Jia et al. found that from 15 months to 30 months after an earthquake, the prevalence of depression ranged from 13.9 to 13.5%, and the prevalence of comorbid PTSD ranged from 4.2 to 4.7% (8).

Few studies have explored the factors that reduce the severity of PTSD over time. Our previous studies indicated that short-term cognitive behavioral therapy (CBT) group interventions reduced PTSD symptoms and depression in bereaved adolescents following the Wenchuan earthquake (16). Yule also reported that girls who survived the accident on the cruise ship Jupiter experienced improvements in PTSD symptoms through CBT that persisted 9 months after treatment (17). In addition to psychotherapy, resilience is also thought to be associated with PTSD mitigation (18). Traumatic experiences can facilitate the development of psychological resilience, which in turn can lead to more effective coping in the aftermath of severe trauma.

A number of demographic, psychosocial, and objective trauma-related risk factors for the development of PTSD have been identified in bereaved children and adolescents after earthquakes. However, few studies have explored the factors that influence changes in PTSD symptoms. This study aimed to investigate (1) the risk factors affecting PTSD symptoms 2 years after the earthquake and (2) the factors affecting changes in symptoms through a 1-year follow-up study.

## Method

### Study Participants

The study was conducted 2 years after the earthquake. A total of 203 children and adolescents from two schools in Beichuan and Shifang were initially recruited to participate in this study. Both schools were at the epicenter of the Wenchuan earthquake, and approximately 1/3 of their students died. None of the participants had received mental health treatment prior to this study. The Institutional Review Board of the Mental Health Centre of Western Hospital and the Ministry of Education of Beichuan approved this study. The parents and schools provided written informed consent.

For the 1-year follow-up study, we contacted 151 children and adolescents (74%). The other students had graduated or left the school without leaving their contact information.

### Measures

The Chinese version of the Children's Revised Impact of Event Scale (CRIES-13) was used to assess PTSD symptoms in the adolescents (19). The scale was designed for children older than 8 years who have experienced a traumatic event and can read independently. The CRIES-13 was translated into Chinese by repeated translation and back translation; the iterations took into consideration the local Chinese culture. The 13-item scale covers 13 symptoms, and each symptom can be rated as one of four levels (from 0 to 5) according to the severity: 0 = no, 1 = rarely, 3 = sometimes, 5 = often. The total scores ranges from 0 to 65 points. The scale has three factors. The first factor is the flashback factor (items 1, 4, 8, and 9). The second factor is the avoidance numbness factor (items 2, 6, 7, and 10). The third factor is the high level of vigilance (items 3, 5, 11, 12, and 13). Children who are impacted more severely by traumatic experiences have higher scores. In a pilot study of 252 students in grades 3, 4, 5, 7 and 8 in schools in Hanwang and Yingxiu, stratified random cluster sampling was conducted to examine the reliability and validity of the CRIES-13. This pilot study confirmed that the CRIES-13 had good reliability and validity (Cronbach's α = 0.903). When the cut-off point of 18 points on the CRIES-13 was chosen to screen for PTSD, the Youden index (57.6%) was maximized, the sensitivity was 81.1%, the specificity was 76.5%, and the diagnostic accuracy was 81.1%.

Depressive symptoms were assessed with the Center for Epidemiologic Studies Depression Scale (CES-D) (20). The scale was designed to measure the current level of depressive symptomatology, with an emphasis on the affective component and depressed mood. Scores range from zero to 60, with higher scores indicating more symptoms (Cronbach's α = 0.912 in this study).

Psychological resilience was measured with the Connor-Davidson Resilience Scale (CD-RISC) (21). The scale has 25 items investigating five factors: personal competence, tolerance of negative affect, acceptance of change, sense of internal control and spirituality. Scores range from zero to 100, with higher scores indicating more resilience (Cronbach's α = 0.937 in this study).

### Treatment

In total, 58 adolescents with CRIES-13 scores greater than 32 received CBT. CBT interventions were based on the manual “Children and Disasters: Teaching Recovering Techniques,” which integrates principles from CBT, eye movement desensitization reprocessing (EMDR), and anxiety control training. It has successfully helped child victims in previous earthquakes. Each student participated in a number of 1-h sessions (range 4–10, mean = 6). All of them were taught to use relaxation techniques, deal with nightmares, and control the intrusion of painful images, and they were exposed to triggers gradually.

### Statistical Analysis

The predictors of PTSD severity at study entry were assessed with hierarchical linear regression analysis with the forced entry of three blocks of covariates: demographics as the first block, disaster event-related factors as the second block, and depression and resilience as the third block. The predictors of changes in PTSD severity from study entry to follow-up were also evaluated with hierarchical linear regression analysis with the forced entry of three blocks of covariates: CBT treatment was entered in the last block to specifically test whether this factor predicted changes *in PTSD* severity scores after considering family support and changes in depression and resilience. The changes in scores were calculated by subtracting the study entry scores from the follow-up scores.

## Results

At study entry, there were 80 boys and 123 girls, with a mean age of 11.91 years. Approximately 30% of the children had lost at least one family member during the earthquake. Approximately 6.9% of the children had been buried. Approximately 12.3% of the children had been injured during the earthquake. In addition, 46.3% of the children witnessed other people being injured or suffering. Approximately 35% of the children witnessed death. Almost all children had several symptoms of PTSD and depression, with a mean CRIES score of 30.22 and a mean CES-D score of 22.37. In the follow-up study, nearly 66.2% of the children still had some PTSD symptoms, with a mean CRIES score of 23.56 and a mean CES-D score of 13.85 (Table 1).

**Table 1:** Description of the sample population.

**Variable**	**Study entry (*n* = 203)**	**1 year follow-up (*n* = 151)**
Male gender	80 (39.4%)	62 (41.1%)
Mean age, years (range 8–16)	11.91	10.85
Family member killed	67 (33.0%)	44 (29.1%)
Child was buried	14 (6.9%)	14 (9.3%)
Child was injured	25 (12.3%)	23 (15.2%)
Child witnessed death	71 (35.0%)	50 (33.1%)
Child witnessed another person be injured/suffer	94 (46.3%)	59 (39.1%)
CBT treatment	0	58 (38.4%)
CRIES	30.22 ± 12.892	23.56 ± 11.285
CRIES score greater than 18	80.8%	66.2%
CES-D	22.37 ± 14.517	13.85 ± 10.943
CD-RISC	53.71 ± 14.03	56.90 ± 13.953

### Predictors of PTSD Severity at Study Entry

Table 2 shows the results of the hierarchical linear regression analysis with three blocks of predictors of PTSD severity at study entry. In model 1a, older age significantly predicted a greater severity of PTSD and explained a significant 4.1% of the variance. In model 1b, family member death and child burial but not older age emerged as significant predictors and explained a significant 21.9% of the variance. In the final model, 1c, family member death and child burial were also significant predictors, and depression emerged as a significant predictor, explaining a significant 16.7% of the variance (Table 2).

**Table 2:** Predictors of PTSD severity at study entry.

**Model statistics**	**Entered variables**	***B***	**S.E**.	**β**	***P***
Model 1a *F*_2,202_=5.273, *p* = 0.006, adjusted *R*^2^ = 0.041	Gender	0.384	1.821	0.015	0.833
	Age	1.368	0.421	0.225	0.001
Model 1b *F*_7,202_ = 9.305, *p* = 0.000, adjusted *R*^2^ = 0.223	Gender	−0.009	1.646	0.000	0.995
	Age	0.356	0.483	0.058	0.461
	Family member killed	8.926	2.123	0.326	0.000
	Child was buried	10.991	3.802	0.217	0.004
	Child was injured	2.464	2.937	0.063	0.402
	Child witnessed death	2.080	1.955	0.077	0.289
	Child witnessed another person be injured/suffer	1.842	2.006	0.071	0.360
Mode 1c *F*_9,202_=15.379, *p* = 0.000, adjusted *R*^2^ = 0.390	Gender	1.051	1.466	0.040	0.474
	Age	−0.417	0.452	−0.068	0.358
	Family member killed	5.512	1.970	0.202	0.006
	Child was buried	8.382	3.409	0.165	0.015
	Child was injured	1.021	2.626	0.026	0.698
	Child witnessed death	1.922	1.738	0.071	0.270
	Child witnessed another person be injured/suffer	0.434	1.809	0.017	0.811
	Resilience	−0.084	0.054	−0.092	0.121
	Depression	0.413	0.063	0.465	0.000

Table 3 shows the results of the hierarchical linear regression analysis with three blocks of predictors of the change in PTSD severity from study entry. In model 2a, family support significantly predicted a change in PTSD severity and explained a significant 3.3% of the variance. In model 2b, changes in the scores for resilience and depression significantly predicted a change in PTSD severity and explained a significant 18.7% of the variance. Family support was also a significant predictive factor. In the final model, 2c, changes in the scores for resilience and depression were significant predictors, and CBT emerged as a significant predictor, explaining a significant 33.6% of the variance.

**Table 3:** Predictors of changes in PTSD severity from study entry to follow-up.

**Model statistics**	**Entered variables**	***B***	**S.E**.	**β**	***P***
Model 2a *F*_1,150_=6.163, *p* = 0.014, adjusted *R*^2^ = 0.033	Family support	4.261	1.717	0.199	0.014
Model 2b *F*_3,150_=15.303, *p* = 0.000, adjusted *R*^2^ = 0.222	Family support	3.253	1.548	0.152	0.037
	Change in resilience score	−0.255	0.084	−0.231	0.003
	Change in depression score	0.402	0.097	0.315	0.000
Model 2c *F*_4,150_=48.410, *p* = 0.000, adjusted *R*^2^ = 0.558	Family support	0.747	1.190	0.035	0.531
	Change in resilience score	−0.146	0.064	−0.132	0.025
	Change in depression score	0.212	0.076	0.166	0.006
	CBT	−18.717	1.762	−0.627	0.000

## Discussion

In this study, we examined the risk factors for PTSD 2 years after the Wenchuan earthquake and the factors predictive of reductions in PTSD symptoms at the 1-year follow-up. The results showed that 80% of children still had PTSD symptoms 2 years after the Wenchuan earthquake, and 66.2% of the children had PTSD symptoms that persisted for 3 years. Previous studies have also indicated that PTSD can last a long time, even many years and that some symptoms can appear a few years after the traumatic event (8). Karakaya et al. screened students for symptoms of PTSD three and a half years after the Marmara earthquake and found that 60.5% of the students still had moderate or more severe symptoms, which was consistent with our study (22). The prevalence of PTSD in students ranged from 5.7 to 10.7% 36 months after the Wenchuan earthquake, which was lower than the prevalence reported in this study (23). This may be because (1) the aim of this study was to explore the symptoms of PTSD, while the other study identified the prevalence of PTSD, and (2) the subjects in this study lost more family members and experienced more burial and injury than the subjects in the other study.

In this study, the objective trauma characteristics of the loss of family members and being buried were significant predictors of PTSD severity, and they explained a significant 21.9% of the variance. These findings were in accord with those of previous studies, which demonstrated that children with more severe exposure to the traumatic event had more PTSD symptoms (24, 25). The loss of family members affects the emotional attachment system in children. Some children feel guilty because they are unable to rescue their families. This could promote the development of PTSD in children.

We also found that depressive symptoms were a significant predictor of PTSD. Previous studies have indicated that PTSD and major depressive disorders have a high prevalence of comorbidities, ranging from 48 to 55% (15). During stressful events, individuals with depression are generally more sensitive to the effects of stress, have more intense reactions, and report more negative emotional experiences; these factors may predispose adolescents to developing PTSD. PTSD may also lower resistance to depression. Therefore, the relationships among PTSD, depression and negative life events should be considered in future studies.

A number of meta-analyses and systematic reviews have supported the effectiveness of psychological treatments, especially CBT, for people with PTSD (26). Our previous randomized controlled studies also suggested that CBT was effective at reducing PTSD symptoms (27, 28). In this follow-up study, we found that CBT emerged as a significant predictor of a reduction in PTSD symptoms, explaining a significant 33.6% of the variance. In addition, reducing depression symptoms and increasing resilience can also reduce PTSD severity, and these factors explained a significant 18.7% of the variance. Resilience is the dynamic ability to adapt successfully in the face of trauma or a significant threat, and it includes emotion regulation, cognitive flexibility and reappraisal, the ability to experience positive emotions, and the ability to actively obtain social support (29). During stressful events, resilient individuals can notice negative thoughts, replace them with a more positive perspective and decrease their level of autonomic arousal to actively cope with the stress. Previous studies stated that emotional coping is a risk factor for psychological problems, especially suicidal ideation, in adolescent survivors of the earthquake in L' Aquila (30). Individuals with resilience can buffer stressors by applying positive emotional coping skills after traumatic exposure (31). All of these factors contribute to the reduction in PTSD symptoms.

## Limitations

First, the children in this study had relatively more severe exposure to the earthquake, and they may not be representative of children in general. Second, the measures in this study relied on self-reported data rather than structural interview data, which may have caused reporting bias. Third, the sample size was too small to generalize the present results. Fourth, a decrease in the number of follow-ups also has bias errors on the predictors of changes in PTSD severity. Fifth, previous traumatic events (such as other childhood traumas) and other current psychopathological comorbidities, such as mood disorders, anxiety disorders or alcohol and substance abuse, which might influence PTSD and depressive symptoms, need to be considered in future studies. Last, the mental state of the children before the earthquake was not investigated, and it cannot be concluded that there is a causal relationship between PTSD and depression.

In summary, the loss of family members and being buried during the earthquake are strong risk factors for PTSD in adolescents 2 years after an earthquake. In addition to using psychological interventions to improve PTSD symptoms, improvements in depression and resilience should also be considered.

## Data Availability Statement

The original contributions presented in the study are included in the article/supplementary material, further inquiries can be directed to the corresponding author/s.

## Ethics Statement

The studies involving human participants were reviewed and approved by The Institutional Review Board of the Mental Health Centre of Western Hospital and the Ministry of Education of Beichuan. Written informed consent to participate in this study was provided by the participants' legal guardian/next of kin.

## Author Contributions

CL and HD conceptualized and designed the work. YC, HD, and KK collected data. YC and CL analyzed data and performed statistics. YC drafted the manuscript, with critical comments from CL and HD. All authors reviewed the manuscript and approved the final version for submission.

## Funding

This work was supported by the Youth Foundation, Hong Kong (YF2012B-HX01).

## Conflict of Interest

The authors declare that the research was conducted in the absence of any commercial or financial relationships that could be construed as a potential conflict of interest.

## Publisher's Note

All claims expressed in this article are solely those of the authors and do not necessarily represent those of their affiliated organizations, or those of the publisher, the editors and the reviewers. Any product that may be evaluated in this article, or claim that may be made by its manufacturer, is not guaranteed or endorsed by the publisher.
